# Mechanism of Inosine from *Lactiplantibacillus plantarum* MWFLp-182-Treated Mice Model in Alleviating D-Galactose-Induced HT-22 Cell Injury via Oxidative and Inflammatory Pathways

**DOI:** 10.3390/foods15020349

**Published:** 2026-01-18

**Authors:** Jianbo Tang, Qing Zhao, Hanying Tan, Ni Yang, Qun Yu, Zhiyu Cui, Xiaochun Li, Yanghe Luo, Guangqing Mu, Xiaomeng Wu, Hui Nie

**Affiliations:** 1 Guangxi Key Laboratory of Health Care Food Science and Technology, Hezhou University, Hezhou 542899, China; startar2020@163.com (J.T.);; 2 Guizhou Food Processing Institute, Guizhou Academy of Agricultural Sciences, Guiyang 550006, China; 3 Guizhou Key Laboratory of Agricultural Biotechnology, Guiyang 550006, China; 4 School of Food Science and Technology, Dalian Polytechnic University, Dalian 116034, China; 5 State Key Laboratory of Food Science & Resources, Jiangnan University, Wuxi 214122, China; 6 Department of Agricultural, Food and Nutritional Science, University of Alberta, Edmonton, AB T6G 2P5, Canada

**Keywords:** inosine, HT-22 cells, oxidative, apoptosis, inflammatory

## Abstract

Gut microbial metabolites play a crucial role in modulating cognitive function. In a previous animal study, oral administration of *Lactiplantibacillus plantarum* MWFLp-182 (*L. plantarum* MWFLp-182) significantly increased inosine levels in both the serum and feces of D-galactose (D-gal)-induced mice, which was accompanied by improved cognitive performance. Building on this finding, we further investigated the neuroprotective mechanisms of inosine derived from *L. plantarum* MWFLp-182 in alleviating D-gal-induced neuronal damage in HT-22 cells. Reverse transcription-quantitative PCR (RT-qPCR) was used to analyze the addition of inosine (250 μg/mL, 500 μg/mL), which considerably reduces oxidative stress induced by D-gal (20 mg/mL), on the regulation of mRNA expression of the nuclear factor erythroid 2-related factor (*Nrf2*)/hemeoxygenase 1 (*HO-1*) signaling pathway factors. Compared to the D-gal group, the inosine-treated group exhibited a 4.3-fold and 8.7-fold increase in *HO-1* and *Nrf2* levels, respectively. Furthermore, inosine alleviates neuroinflammation by modulating the mRNA expression of the Toll-like receptor 4 (*TLR4*)/myeloid differentiation primary response protein 88 (*MyD88*)/nuclear factor kappa B (*NF-κB*) signaling pathway. Compared to the D-gal group, the inosine-treated group showed reductions of 41.75%, 28.29%, and 32.17% in *TLR4*, *MyD88*, and *NF-κB* levels, respectively. Moreover, immunofluorescence staining revealed that inosine exhibits anti-apoptotic properties by enhancing the levels of neurotrophic factors, including nerve growth factor (NGF) and brain-derived neurotrophic factor (BDNF), while simultaneously lowering the expression of the pro-apoptotic protein bcl-2-associated X (*Bax*). These findings suggest that inosine, a differentially expressed metabolite identified in a probiotic-intervention mouse model, alleviates D-gal-induced neuronal damage in HT-22 cells by modulating oxidative, inflammatory, and apoptotic pathways, providing mechanistic insights into the neuroprotective effects of this metabolite.

## 1. Introduction

The global population aged 65 years and older is expected to rise to 2 billion by 2050 [1,2]. Neurodegenerative diseases associated with aging have become increasingly prominent [2]. Among these, cognitive impairment is particularly notable. It encompasses attentional deficits, memory decline, and related domains. This condition has emerged as a critical public health challenge, severely compromising the quality of life of affected individuals. Alzheimer’s disease (AD), characterized by a progressive and irreversible decline in memory and other higher level cognitive functions, is the most common neurodegenerative disorder [3]. Neural oxidative damage and neuroinflammation are key drivers of cognitive decline [3,4]. In particular, increased levels of reactive oxygen species (ROS) and proinflammatory cytokines, such as interleukin-1β (IL-1β) and tumor necrosis factor-α (TNF-α), activate neurotoxic pathways mediated by microglia. This activation accelerates neuronal damage and underlies the development of cognitive impairment [5].

Recently, gut metabolomic analysis has emerged as a key approach for biomarker discovery. Moreover, metabolites are critical biomarker indicators that help assess the pathophysiology of health and disease. Microbial molecules can be used to treat cognitive impairments. Probiotics produce antioxidant and anti-inflammatory effects by modulating the gut microbiota and their metabolites. This action preserves the intestinal barrier and improves cognitive function [6,7,8,9]. Gut-derived metabolites, such as short-chain fatty acids (SCFAs) and serotonin (5-HT), pass through the blood–brain barrier (BBB) and protection of neural cells [7]. *Lactobacillus plantarum* CCFM405 modulates brain function and behavior by remodeling the gut microbiota structure to enhance the biosynthesis of amino acids, particularly branched-chain amino acids (BCAAs), which cross the BBB via amino acid transporters and enter the central nervous system (CNS) [7]. The concentrations of *Lactobacillus* and *Akkermansia* in the gastrointestinal tract were positively correlated with lithocholic acid (LCA) levels. According to a previous study, LCA boosts the activity of sirtuin, a member of the deacetylase family that is related to longevity. This activates AMP-activated protein kinase (AMPK) [10,11]. Zha et al. revealed that lysophosphatidylcholine (LPC), a metabolite derived from *Bacteroides* ovatus, alleviated cognitive impairment by suppressing ACSL4 expression via GPR119, thereby inhibiting ferroptosis, improving cognitive function, reducing neuroinflammation, and mitigating myelin degeneration [10]. Notably, reduced levels of *Bacteroides* and LPC have been observed in patients with cognitive impairment, suggesting that targeting the *Bacteroides*-LPC-GPR119-ferroptosis signaling axis may offer therapeutic strategies for cognitive disorders [12].

*Lacticaseibacillus rhamnosus* can biosynthesize inosine, a bioactive purine nucleoside integral to energy metabolism, neuronal signaling modulation, and redox homeostasis [13]. Recent studies have highlighted its potential for neuroprotection (Khanal et al. 2025) and cognitive enhancement [14,15,16]. Research demonstrated that inosine effectively ameliorates oxidative stress in cognitively impaired mice by suppressing lipid peroxidation, nitrite, and TNF-α levels, while concurrently restoring the activity of key antioxidant enzymes reduced glutathione peroxidase (GSH-PX) and superoxide dismutase (SOD) [17]. Together, these influences improve memory and learning abilities (Ruhal et al. 2018) through antioxidant (Teixeira et al. 2022) and anti-inflammatory mechanisms, in addition to promoting neuronal survival within the CA1 area of the hippocampus [13,14,18].

In the current study, the oral administration of *L. plantarum* MWFLp-182 significantly increased inosine concentrations in both the feces and serum of D-gal-treated mice with cognitive impairment. To assess the effects of inosine on cognitive function, we conducted antioxidant, anti-inflammatory, and anti-apoptotic assays, reverse transcription-quantitative PCR (RT-qPCR), and immunofluorescence. Our findings offer a solid theoretical foundation for understanding how inosine can alleviate impairments in HT-22 cells. Additionally, this study introduces a novel compound that may contribute to D-gal-induced senile neuronal damage and cognitive impairment-related phenotypes.

## 2. Materials and Methods

### 2.1. Materials

D-gal, bought from Sigma-Aldrich (St. Louis, MO, USA), and inosine (Beijing Solarbio Science & Technology Co., Ltd., Beijing, China) were used in study. The scientist obtained antibodies used in the study, namely NGF and BDNF, from Beyotime Biotechnology Co., Ltd. (Shanghai, China).

### 2.2. Animals and Treatments [6]

The experimental animals were 7-week-old male BALB/c mice, which were bred by Liaoning Changsheng Biotechnology Co., Ltd. (Benxi, China). The experiments were approved by the Animal Ethics Committee of Dalian Polytechnic University. The procedures were performed in accordance with the Guide for the Care and Use of Laboratory Animals of Dalian Polytechnic University (approval number: DLPU2024001). The procedures involving the animals and treatments followed methods that have been outlined in prior works [6]. The control group (LK) received intraperitoneal injections of physiological saline and was also administered physiological saline orally by gavage. The model group (LD) received intraperitoneal injections of 800 mg/kg body weight D-gal daily and was orally gavaged with physiological saline. The treatment group (LH) was injected intraperitoneally daily with D-gal with a dose of 800 mg/kg body weight, and *L. plantarum* MWFLp-182 was orally gavaged with 1 × 10^9^ CFU/mL per mouse. After culturing the bacteria for 16–18 h, centrifuging at 4000× *g* for 5 min, harvesting the precipitate, and resuspending it in physiological saline three times, the final concentration was adjusted to 1 × 10^9^ CFU/mL. Each mouse received 0.2 mL of the solution. The experiment lasted for 8 weeks, after which mouse serum and feces were collected for metabolomics analysis.

### 2.3. Serum Metabolomic Analysis

A serum metabolomic analysis was performed based on previously published methodologies, incorporating slight adjustments [19]. The serum samples obtained by centrifugation of mouse blood were mixed with a methanol and acetonitrile solution containing 2% L-2-chlorophenylalanine, with methanol and acetonitrile accounting for 50% each. After vortexing for 30 min, the samples were ultrasound-assisted extracted at 40 kHz for 30 min at 5 °C and incubated at −20 °C for 30 min. Subsequently, the sample was centrifuged at 13,000× *g* for 15 min, and the supernatant was dried with nitrogen and dissolved in 50% acetonitrile solution. The reconstituted mixture was shaken for 30 min and sonicated for 5 min at 4 °C, the supernatant was centrifuged again, and the mixture was filtered through a 0.22 μm filter and finally transferred to an autosampler vial. The chromatographic analysis was performed using an ACQUITY UPLC HSS T3 column (100 mm × 2.1 mm, 1.8 µm; Waters, Milford, MA, USA) maintained at 40 °C. The mobile phase consisted of acetonitrile, water, and isopropanol, and gradient elution was performed at a flow rate of 0.4 mL/min and an injection volume of 3 μL, followed by mass spectrometry detection.

### 2.4. Fecal Metabolomic Analysis

Fecal metabolomic analysis was conducted using methods previously studied, with minor modifications [1]. Approximately 60 mg of fecal sample was placed in a 1.5 mL centrifuge tube containing tissue grinding steel beads and 600 µL of methanol and water. Then the sample was homogenized with a tissue grinder at a frequency of 60 Hz for 2 min. The samples were combined and placed at −40 °C overnight, followed by centrifugation at 13,000× *g* for 10 min. Finally, 200 μL of supernatant was aspirated and dried by nitrogen. The dried sample was resuspended in 300 μL of methanol-water (1:4, *v*/*v*) solution, sonicated in an ice water bath for 3 min, and allowed to stand at −40 °C for 2 h, and centrifuged again for 10 min (13,000× *g*, 4 °C). A total of 150 μL of the supernatant was drawn using a syringe, passed through a 0.22 μm organic phase syringe filter, and transferred into an LC vial. Samples were stored at −80 °C before analysis by UPLC-MS, using a Waters ACQUITY UPLC I-Class Plus and a Thermo Q Exactive mass spectrometer. The separation of the above target compounds was performed using an ACQUITY UPLC HSS T3 column (100 mm × 2.1 mm, 1.8 µm; Waters, Milford, MA, USA) with column temperature 45 °C. The mobile phase consisted of methanol, acetonitrile and water, and gradient elution was performed, the injection volume was 2 μL, and the flow rate was maintained at 0.35 mL/min. Mass spectrometry detection was performed in negative ion mode.

### 2.5. Cell Cytotoxicity Assay

The CCK-8 assay was performed according to previous studies [20]. Mouse hippocampal HT-22 cells were cultured in DMEM medium (Gibco, Grand Island, NY, USA) containing 10% (*v*/*v*) heat-inactivated fetal bovine serum and 1% (*v*/*v*) penicillin-streptomycin. The cells were seeded in 96-well plates at a density of 5 × 10^3^ cells per well and incubated at 37 °C for 24 h. After washing with PBS three times, the HT-22 cells were treated with 100 μL of inosine at various concentrations (50, 100, 200, 300, 400, 500, and 600 μg/mL), and different concentrations of D-gal (10, 20, 30, 40, 50, 60, 70, 80, 90, and 100 mg/mL) were added to the cell culture, respectively. DMEM medium (10% FBS) was used as a blank control. After 24 h of being incubated, each well will have 10% CCK-8 reagent added to it according to the manufacturer’s instructions. The wells will then be incubated for 30 to 60 min. A microplate reader (Multiskan GO 1510; Thermo, Waltham, MA, USA) measured the absorbance at 450 nm. Cell viability was calculated using the formula:

Cell viability (100%) = (Adrug − Ablank)/(Acontrol − Ablank) × 100%.

### 2.6. Measurement of Intracellular ROS in HT-22 Cells

Intracellular ROS levels in HT-22 cells were measured based on the protocol described by Nie et al. [21]. HT-22 cells were seeded in a 24-well plate at a density of 1 × 10^5^ cells per well at 37 °C for 24 h. At 85% confluence, the cells were washed three times with PBS and HT-22 cells were treated with D-gal (0.5 mL; 20 mg/mL) and pretreated with inosine at different concentrations (0.5 mL; 0, 250, or 500 μg/mL) for 24 h. The cells were washed three times with PBS, and incubated with 500 μL of 10 mmol/L DCFH-DA in the dark at 37 °C for 30 min. Subsequently, the cells were washed twice with PBS and the fluorescence intensity was determined using fluorescence microscopy (OLYMPUS DP74, Japan) at excitation and emission wavelengths of 488 and 525 nm, respectively.

### 2.7. Determination of mRNA Expression

In this study, RT-qPCR was used to quantify the mRNA expression levels of various factors, including the expression levels of cytokines *IL-1β*, *IL-10*, *TNF-α*, *Nrf2*, *HO-1*, *Bax*, and *Bcl-2* [21]. In addition, the expression levels of *AKT*, *Caspase-3*, *Caspase-9*, *MyD88*, *NF-κB*, and *TLR4* were also detected. At the same time, the expression of *BDNF* and *NGF* was also evaluated. A detailed list of the selected genes and primers for RT-qPCR is provided in Table S1.

### 2.8. Immunofluorescence Staining

Immunofluorescence staining was carried out with slight modifications to previously described protocols [22,23]. HT-22 cells were co-cultured in 12-well plates at a density of 5 × 10^4^ cells per well with D-gal (0.5 mL; 20 mg/mL) and pretreated with inosine at different concentrations (0.5 mL, 0, 250, or 500 μg/mL) for 24 h. Subsequently, cells were washed once with PBS and fixed with 4% paraformaldehyde for 10 min at room temperature (RT) and permeabilized with 0.1% Triton X-100 in PBS. Cells were then blocked with 3% bovine serum albumin (BSA) in PBS for 1 h.

Secondary antibodies for BDNF or NGF were added and incubated overnight at 4 °C. FITC-labeled goat anti-rabbit IgG secondary antibody was added and incubated for 1 h at room temperature. Subsequently, nuclear staining was performed, which contained DAPI. Fluorescence microscopy was used for detection, with an excitation wavelength of 330–380 nm and an emission wavelength of 420 nm. FITC was excited at 488 nm, and an emission wavelength of 500–540 nm was observed.

### 2.9. Statistical Analysis

All the experiments were performed multiple times and the data were given as mean standard deviation. Statistical analysis was performed with the help of IBM SPSS Statistics 23. Differences in mRNA and protein expression levels among groups were evaluated by one-way ANOVA, followed by post hoc comparisons using the Least Significant Difference (LSD) test. A *p*-value < 0.05 was considered statistically significant.

## 3. Results

### 3.1. Serum Metabolite Analysis

**Figure 1:**
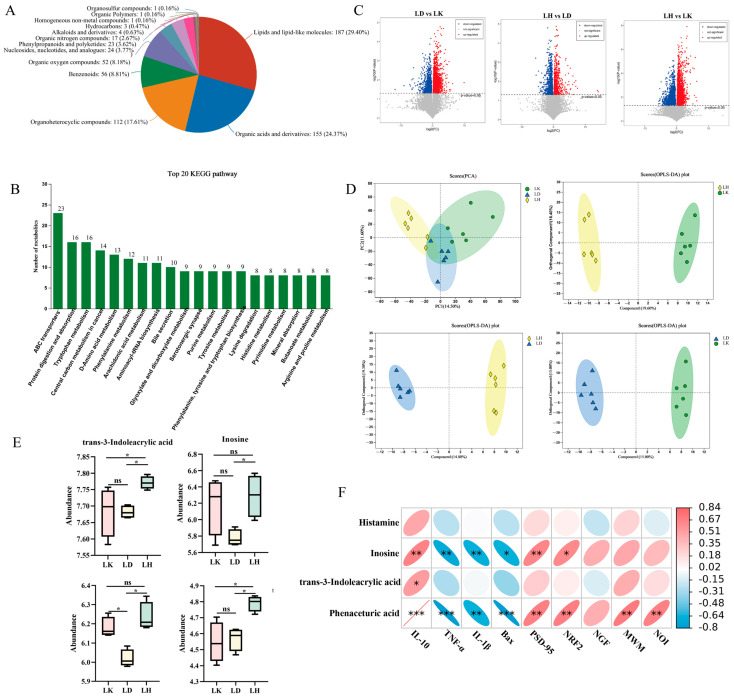
The metabolites and KEGG function classification (**A**–**C**). Volcano diagram of differential metabolite analysis of the LD, LH and LK. Blue dots represent downregulated differentially expressed metabolites, red dots represent upregulated differentially expressed metabolites, and gray represents insignificant expressed metabolites. (**D**) PCA and OPLS-DA were employed to compare the metabolic profiles among different groups. (**E**) trans-3-indoleacrylic acid, inosine, phenaceturic acid and histamine are metabolized in serum among different groups. Data are presented as mean ± SD. (**F**) Pearson’s correlation analysis between behavioral data, neurological data, and proinflammatory cytokines of serum. The color intensity and ellipse size show the strength of the correlation, * *p* < 0.05, and ** *p* < 0.01, *** *p* < 0.001. Red color represents a pons indicates no significant difference. sitive correlation, whereas blue color represents a positive correlation.

As depicted in Figure 1A, there were 636 metabolites identified from these classes. The metabolites comprise lipids and lipid-like molecules (187, 29.40%), organic acids and derivatives (155, 24.37%), organoheterocyclic compounds (112, 17.61%), benzenoids (56, 8.81%), organic oxygen compounds (52, 8.81%), nucleosides, nucleotides, and analogues (24, 3.77%), phenylpropanoids and polyketides (23, 3.62%), organic nitrogen compounds (17, 2.67%), alkaloids and derivatives (4, 0.63%), hydrocarbons (3, 0.47%), homogeneous non-metal compounds (1, 0.16%), organic polymers (1, 0.16%), and organosulfur compounds (1, 0.16%). The key functional pathways of these metabolites were annotated using KEGG, with the top 20 pathways illustrated in Figure 1B. Among these, “ABC transporters” were the most abundant, followed by “Protein digestion and absorption” and “Tryptophan metabolism.” Volcano plots (Figure 1C) identified 139 differentially expressed metabolites between the LH and LK groups (59 upregulated and 80 downregulated), and 88 differential metabolites between the LD and LK groups (60 upregulated and 28 downregulated). These findings were rigorously analyzed to elucidate the metabolic disparities among the experimental cohorts.

The OPLS-DA models (Figure 1D) showed a clear distinction between the LH and LK groups (R2X = 0.196, R2Y = 0.916, Q2 = 0.704), LH and LD groups (R2X = 0.148, R2Y = 0.927, Q2 = 0.569), and LK and LD groups (R2X = 0.15, R2Y = 0.897, Q2 = 0.543). To delve deeper into the differential metabolites among the groups, all 636 metabolites were systematically annotated and screened using fold change (FC) thresholds (≥2 or ≤0.5) and variable importance in projection (VIP) scores (≥1).

The findings in Figure 1E demonstrate the differential metabolites examined in this study. The LH group showed significantly higher concentrations of trans-3-indoleacrylic acid, inosine, phenaceturic acid, and histamine than the LD group (*p* < 0.05). Correlation analysis was conducted on differential metabolites, including trans-3-indoleacrylic acid, inosine, phenaceturic acid, and histamine, and serum inflammatory cytokines, such as IL-10, IL-1β, and TNF-α; hippocampal proteins, such as Bax, NGF, PSD-95, and Nrf2, and behavioral indices comprising MWM and NOI, to clarify the relationships between these differential metabolites and cognitive function [6,24,25]. As depicted in Figure 1F, inosine showed a positive association with IL-10, PSD-95, MWM, NOI, Nrf2 and NST and showed a negative association with Bax, IL-1β, and TNF-α (*p* < 0.05). The relationships between inosine and NGF, MWM, and NOI showed non-significant positive trends (*p* > 0.05). A significant positive correlation was observed between trans-3-indoleacrylic acid and IL-10 (*p* < 0.05). There was a marked positive correlation among phenaceturic acid, IL-10, PSD-95, and Nrf2 (*p* < 0.05). Additionally, there was a significant negative correlation of phenaceturic acid with Bax, TNF-α, and IL-1β (*p* < 0.05). *L. plantarum* MWFLp-182 modulates the relative levels of trans-3-indoleacrylic acid, inosine, phenaceturic acid, and histamine in serum. This modulation attenuated systemic inflammation, enhanced the expression of cognition-associated proteins, and ameliorated cognitive deficits in mice [6].

### 3.2. Fecal Metabolite Analysis

**Figure 2:**
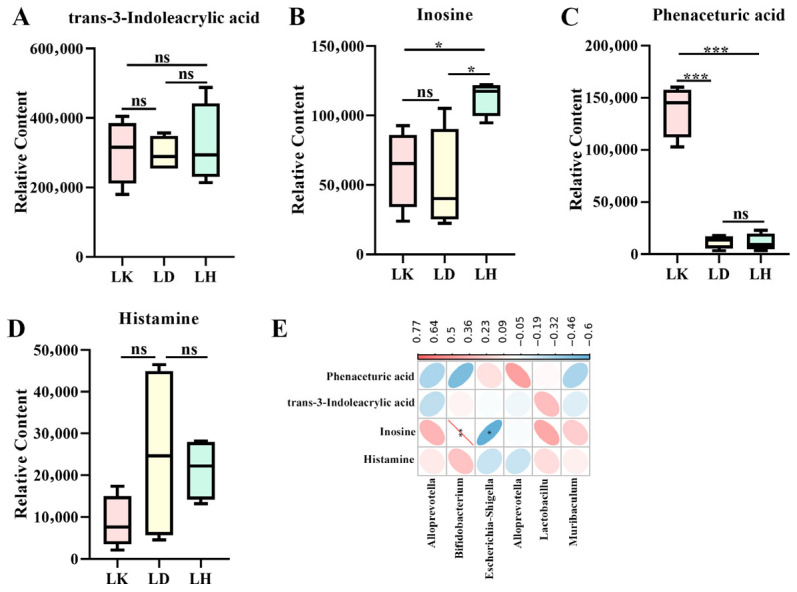
Mouse feces metabolites. (**A**) Trans-3-indoleacrylic acid; (**B**) inosine; (**C**) phenzoylglycine; (**D**) histamineare metabolized; and (**E**) Pearson’s correlation analysis between gut microbiota and metabolite. The color intensity and ellipse size show the strength of the correlation, * *p* < 0.05, ** *p* < 0.01, and *** *p* < 0.001. ns indicates no significant difference. Red color represents a positive correlation, whereas blue color represents a positive correlation.

We further hypothesized that trans-3-indoleacrylic acid, inosine, phenaceturic acid, and histamine present in the serum of mice may translocate through the intestinal barrier into the blood. Consequently, these metabolites were analyzed in the fecal metabolome. As illustrated in Figure 2A–D, there were no notable differences in trans-3-indoleacrylic acid and histamine levels among the three groups (*p* > 0.05). In contrast, phenaceturic acid levels in the LK group were significantly higher than those in the LD and LH groups (*p* < 0.05), whereas inosine levels followed a similar pattern to that in the serum. Considering the involvement of the liver in the metabolism of trans-3-indoleacrylic acid, histamine, and inosine, we suggest that liver processing may explain the inconsistent levels of these compounds in serum and fecal compartments. Spearman’s correlation analysis was conducted between fecal metabolites (trans-3-indoleacrylic acid, inosine, phenaceturic acid, and histamine) and genus-level gut microbiota (*Bifidobacterium*, *Intestinimonas*, *Lactobacillus*, *Alloprevotella*, *Escherichia-Shigella*, and *Muribaculum*) to elucidate interactions between microbial communities and metabolites [6]. As illustrated in Figure 2E, inosine was significantly positively correlated with *Bifidobacterium* (*p* < 0.01) and negatively correlated with *Escherichia-Shigella* (*p* < 0.05). *Lactobacillus* exhibited non-significant positive associations with trans-3-indoleacrylic acid, inosine, and histamine (*p* > 0.05).

### 3.3. Analysis of Cell Viability

**Figure 3:**
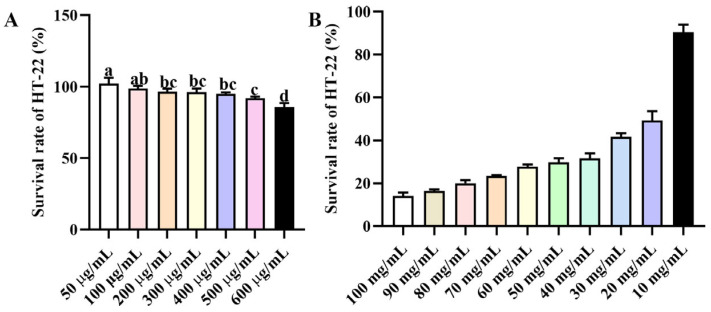
The effect of different components on the viability of HT-29 cells. (**A**) Inosine. (**B**) G-gal. Different lowercase letters (a–d) show significant differences compared with the control (*p* < 0.05), as determined by one-way ANOVA.

Upon conducting an integrated analysis of serum and fecal metabolomic data, inosine was identified as the focal compound. Cytotoxicity was evaluated using the CCK-8 assay in HT-22 cells cultured with varying concentrations of inosine for 24 h. As depicted in Figure 3A, cell viability was maintained at 92 ± 1% and 85.66 ± 2.51% for inosine concentrations of 500 and 600 μg/mL, respectively. Consequently, 500 μg/mL was selected as the working concentration for both compounds in subsequent experiments. The effects of D-gal on cell viability is illustrated in Figure 3B. The results showed that the D-gal dosage affected the toxicity in HT-22 cells. A concentration of 20 mg/mL of D-gal resulted in a cell viability of 49.28 ± 3.8%, which was used for the following experiments.

### 3.4. Effect of Inosine on D-Gal-Induced Oxidative Stress in HT-22 Cells

**Figure 4:**
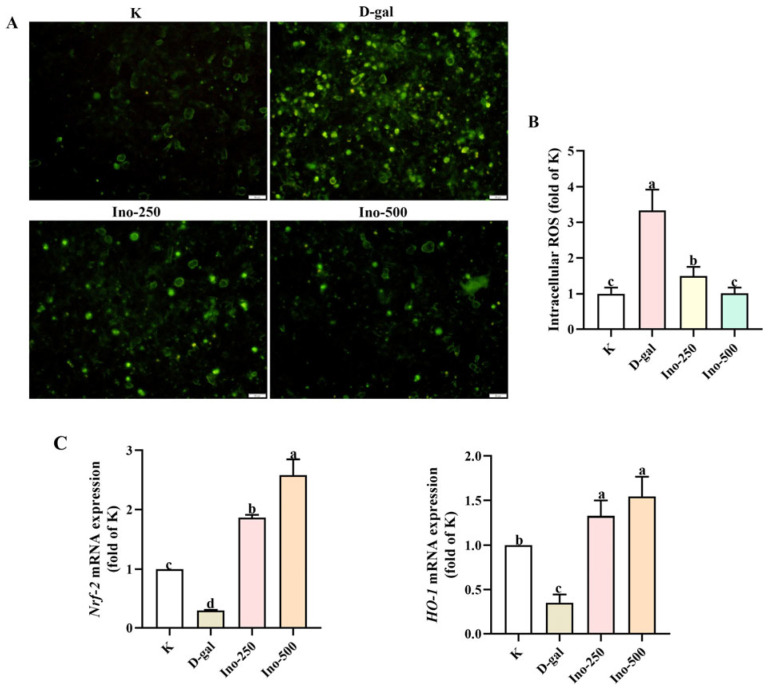
The effect of inosine on D-gal-induced oxidative stress in HT-22 cells. (**A**,**B**) Effects of pretreatment of inosine on the ROS levels of D-gal induced HT-22 cells by fluorescent microscope (20×). (**C**) Effects of inosine on the *Nrf-2* and *HO-1* mRNA expression of HT-22 cells. Data are presented as mean ± SD. Different lowercase letters (a–d) show significant difference compared with the control (*p* < 0.05), as determined by one-way ANOVA.

Signals from fluorescein-labeled cells were analyzed using DCFH-DA to evaluate the intracellular ROS levels. As shown in Figure 4, the fluorescence intensity of the D-gal group was three-fold higher than that of the control group. This confirms that ROS accumulates in HT-22 cells after D-gal induction. Nevertheless, treatment with inosine significantly decreased ROS levels, with the high-concentration group (500 μg/mL: Ino-500) exhibiting notably lower ROS levels than that of the low-concentration group (250 μg/mL: Ino-250). As shown in Figure 4C, D-gal reduced the mRNA expression of *Nrf2* and *HO-1*, which may contribute to the elevated ROS levels and oxidative damage in HT-22 cells. Compared to the D-gal group, the inosine-treated group exhibited a 4.3-fold and 8.7-fold increase in HO-1 and Nrf2 levels, respectively. Based on these findings, it can be concluded that inosine attenuates D-gal-induced oxidative stress by regulating the mRNA expression of factors related to the Nrf2/HO-1 signaling pathway.

### 3.5. Effect of Inosine on D-Gal-Induced Inflammation in HT-22 Cells

**Figure 5:**
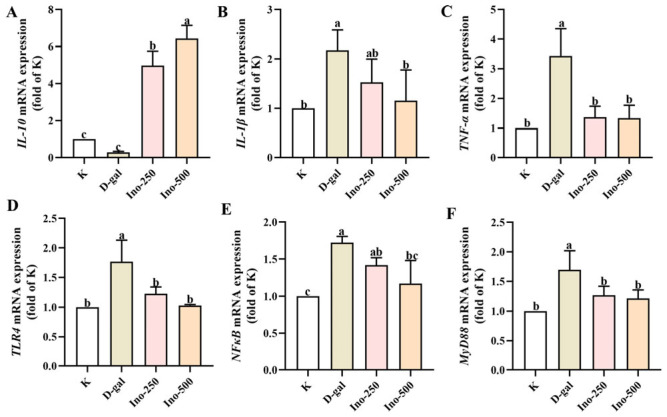
The effects of inosine on mRNA expression of inflammatory cytokines and *TLR4*/*NF-κB* signaling pathway components in HT-22 cells. (**A**) *IL-10*, (**B**) *IL-1β*, (**C**) *TNF-α*, (**D**) *TLR4*, (**E**) *NF-κB*, and (**F**) *Myd88* mRNA expression levels. Data are presented as mean ± SD. Different lowercase letters (a–c) indicate statistically significant differences compared with the control group (*p* < 0.05), as determined by one-way ANOVA.

Excessive activation of glial cells leads to neuroinflammation and neurodegeneration. Therefore, mRNA expression of cytokines (*IL-1β*, *TNF-α*, and *IL-10*) and neuroinflammation-related signaling pathways (*TLR4, NF-κB*, and *MyD88*) were examined in HT-22 cells. As shown in Figure 5A–C, D-gal significantly downregulated the expression of *IL-10* and upregulated the expression of *IL-1β* and *TNF-α* (*p* < 0.05). At the same time, D-gal treatment significantly upregulated the expression of *TLR4*, *MyD88*, and *NF-κB* (Figure 5D–F, *p* < 0.05), regulating the expression of TLR4/MyD88/NF-κB signaling pathway factors, which aggravated neuroinflammation with the destruction of balance. Compared to the D-gal group, the inosine-treated group showed reductions of 41.75%, 28.29%, and 32.17% in *TLR4*, *MyD88*, and *NF-κB* levels, respectively. Furthermore, inosine co-treatment significantly downregulated the expression of *TLR4*, *MyD88*, and *NF-κB* compared to the model group (*p* < 0.05), suggesting that inosine treatment inhibits this pathway and alleviates the D-gal-induced damage to HT-22 cells.

### 3.6. Effect of Inosine on D-Gal-Induced Apoptosis in HT-22 Cells

**Figure 6:**
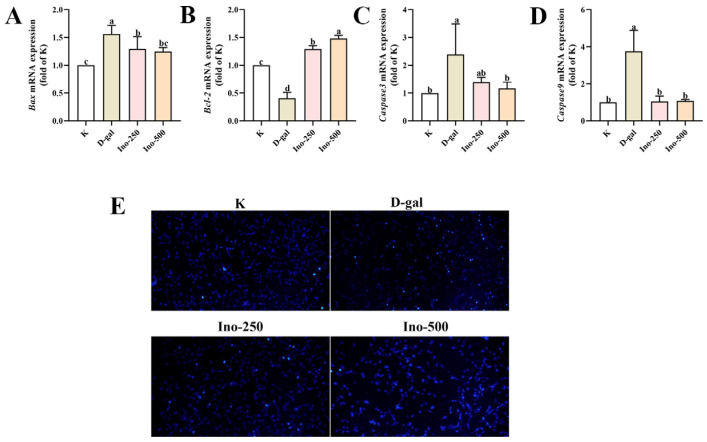
The effects of inosine on apoptosis-related gene expression and D-gal-induced apoptosis in HT-22 cells. Effects of inosine on the *Bax* (**A**), *Bcl-2* (**B**), *Caspase-3* (**C**) and *Caspase-9* (**D**) mRNA expression of HT-22 cells. Data are presented as mean ± SD. Different lowercase letters (a–c) show significant differences compared with the control (*p* < 0.05), as determined by one-way ANOVA. (**E**) The effect of inosine pretreatment on D-gal-induced apoptosis in HT-22 cells (light blue represents apoptotic cells) (20× fluorescence microscopy).

The analysis of apoptosis-related gene expression in D-gal-treated HT-22 cells treated with inosine focused on the mRNA levels of *Bax*, *Bcl-2*, *Caspase-9*, and *Caspase-3*. As shown in Figure 6A–D treatment with D-gal markedly increased mRNA levels of *Bax*, *Caspase-9* and *Caspase-3*, but decreased that of *Bcl-2* (*p* < 0.05), thus inducing apoptosis via the *Bax*-*Caspase-9*-*Caspase-3* pathway. In contrast, co-treatment with inosine (Ino-500) significantly increased *Bcl-2* expression and deceased *Bax*, *Caspase-9*, and *Caspase-3* expression (*p* < 0.05). These results indicate that inosine alleviates D-gal-induced apoptosis in HT-22 cells by regulating the expression of related factors, including reducing the *Bax-Caspase-9-Caspase-3* axis and promoting *Bcl-2*-mediated anti-apoptotic signaling.

Hoechst 33258, a blue fluorescent dye capable of traversing cell membranes, is widely used to detect apoptosis [20]. Hoechst 33258 staining revealed distinct apoptotic patterns in the experimental groups (Figure 6E). After 24 h, the untreated control group exhibited minimal apoptosis, whereas the D-gal-treated group showed extensive apoptosis. Conversely, both inosine-treated groups (Ino-250 and Ino-500) demonstrated significantly reduced apoptotic cell counts, indicating that inosine effectively suppressed D-gal-induced apoptosis in HT-22 cells.

### 3.7. Effect of Inosine on D-Gal-Induced Neurotrophic Factor in HT-22 Cells

**Figure 7:**
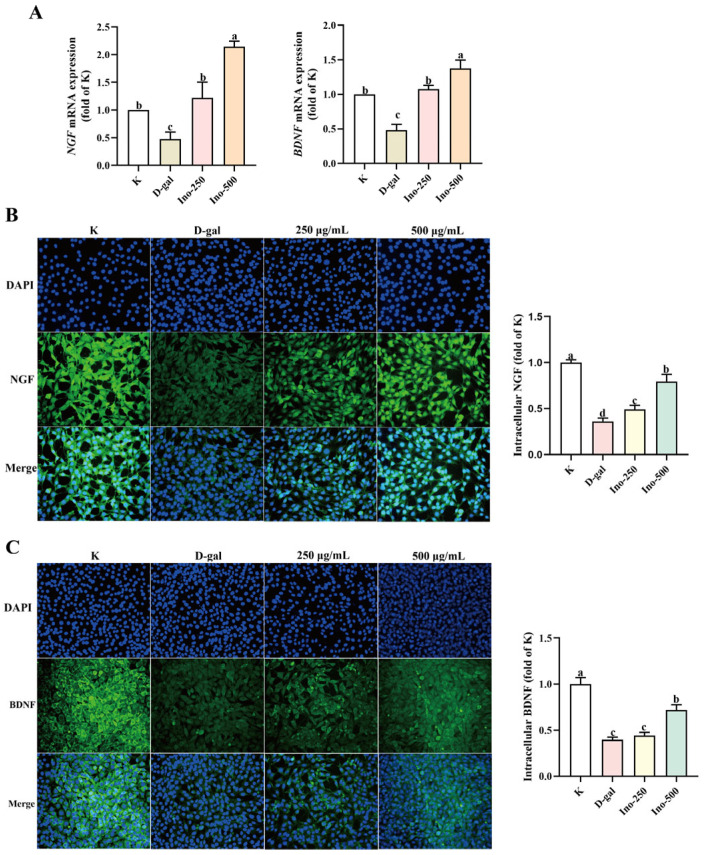
The effects of inosine on neurotrophic factor expression in HT-22 cells. (**A**) The effects of inosine on the *NGF* and *BDNF* mRNA expression of HT-22 cells. (**B**) The effect of inosine on D-gal-induced NGF in HT-2 cells. (**C**) The effect of inosine on D-gal-induced BDNF in HT-2 cells. Data are presented as mean ± SD. Different lowercase letters (a–d) show significant differences.

BDNF and NGF are necessary for the nervous system. They significantly assist in the growth, differentiation, and survival of neurons. Moreover, they assist in the development of memory, learning, and regulation of emotions. RT-qPCR was performed to evaluate *BDNF* and *NGF* mRNA expression in D-gal-treated HT-22 cells. As illustrated in Figure 7A, D-gal significantly decreased the transcription of both neurotrophic factors (*p* < 0.05). In contrast, inosine co-treatment significantly increased BDNF and NGF mRNA levels (*p* < 0.05), suggesting a restorative effect on neurotrophic gene expression.

Immunofluorescence analysis allowed direct visualization of protein-level changes. For NGF (Figure 7B), the D-gal group exhibited a significantly reduced fluorescence intensity (0.36 ± 0.03) compared with that in the control group (K: 1.0 ± 0.03; *p* < 0.05). The inosine-treated groups (Ino-250: 0.49 ± 0.05; Ino-500: 0.79 ± 0.08) showed dose-dependent increases in NGF fluorescence (*p* < 0.05 compared with D-gal), demonstrating mitigation of NGF protein impairment. Similarly, BDNF immunofluorescence (Figure 7C) revealed a decrease in intensity in the D-gal group (0.40 ± 0.03 vs. K: 1.0 ± 0.07; *p* < 0.05), which was reversed by inosine (Ino-250: 0.44 ± 0.04; Ino-500: 0.72 ± 0.06; *p* < 0.05 vs. D-gal), confirming the alleviation of BDNF protein damage.

Immunofluorescence and RT-qPCR data showed that inosine rescued the D-gal-induced inhibition of *BDNF* and *NGF* mRNA and protein expression in HT-22 cells.

## 4. Discussion

D-gal induces oxidative stress and inflammation, leading to HT-22 cell injury [26]. In the present study, *L. plantarum* MWFLp-182 regulated inosine levels in the gut and serum. Additionally, inosine protected against D-gal-induced oxidative stress, inflammation, and apoptosis.

ROS act as key mediators of cellular signaling and participate in numerous physiological processes, including immune responses to microbial and viral challenges, as well as the regulation of cell growth, proliferation, differentiation, survival, aging, and apoptosis [27,28]. Excess intracellular ROS can cause mutations in genes that code for antioxidant defense and DNA repair. These genes are important for mitochondrial DNA stability, and mutations in these genes can lead to increased permeability of the mitochondrial membrane, impaired mitochondrial respiratory function and alterations in calcium homeostasis [29]. Nrf2, a 589 amino acid long redox-sensitive transcription factor and protein with a molecular weight of 66.1 kDa, reduces the oxidative damage caused by oxidative stress by regulating the expression of antioxidant enzymes and helping in the detoxification of ROS [30]. Thus, it helps to maintain cellular redox homeostasis. The heme oxygenase (HO) system includes two catalytically active isoforms: inducible HO-1 and constitutive HO-2. HO isoforms are the key enzymes that degrade heme [31]. These enzymes convert heme into free iron, carbon monoxide (CO), and biliverdin. HO-1 has been highlighted as a cell-protective enzyme that produces biliverdin and CO, which are antioxidant and anti-inflammatory agents. HO-1 activation protects against oxidative stress, apoptosis, and cell death, in both cellular and animal models. In the present study, we investigated the effect of inosine on D-gal-induced oxidative stress in HT-22 cells and found that inosine mitigated D-gal-induced oxidative injury in HT-22 cells.

Neuroinflammation is defined as the activation of the innate immune system in the brain in response to inflammatory triggers and is considered a key characteristic of neurodegenerative disorders [32]. The involvement of hyperactivated glial cells in promoting neuroinflammation and neurodegeneration has been extensively studied. Additionally, proinflammatory cytokine expression is increased (IL-6, IL-1β, and TNF-α) as is anti-inflammatory cytokines (IL-10) expression, causing a correlation with microglial activation [33]. In this study, we investigated how inosine affects inflammatory damage in a D-gal-induced model. The TLR signaling pathway initiates and activates inflammatory responses in macrophages [34]. When TLR recognizes its ligand, the downstream signaling pathway is activated by MyD88, including mitogen-activated protein kinase (MAPK) and transcription factor NF-κB, promoting the production of proinflammatory cytokines. After inosine intervention, the expression levels of anti-inflammatory cytokine *IL-10* increased, and the expression levels of proinflammatory cytokines *IL-1β* and *TNF-α* decreased in HT-22 cells pretreated with D-gal (Figure 5). These results indicate that the expression of factors related to the *TLR4/MyD88/NF-κB* signaling pathway is inhibited under the action of inosine, thereby alleviating cellular inflammation induced by D-gal.

Inflammation and oxidative stress trigger apoptosis, a key form of programmed cell death, whose dysregulation is closely associated with the onset and progression of various diseases [35]. The anti-apoptotic protein, Bcl-2, regulates cytochrome C release from the mitochondria and modulates interactions among apoptotic factors such as Caspase-9 and Bax. Bax, a pro-apoptotic member of the Bcl-2 family and recognized tumor suppressor, promotes apoptosis [36]. Oxidative stress activates the Bax–Caspase-9–Caspase-3 pathway, which initiates apoptotic cell death [37,38]. In the present study, inosine treatment decreased *Bax*, *Caspase-9*, and *Caspase-3* mRNA levels and upregulated *Bcl-2* expression in HT-22 cells, thereby alleviating D-gal-induced cognitive impairment in mice (Figure 6) [39]. It should be noted that the mechanistic explanation in this study is mainly based on transcriptional changes detected by RT-qPCR. Changes in apoptosis-related genes (*Bax*, *Bcl-2*, *Caspase-9*, and *Caspase-3*) as well as oxidative stress and inflammation-related pathways will be detected in the future using Western blot or ELISA.

BDNF and NGF play crucial roles in the nervous system by supporting neuronal growth, differentiation, and survival, as well as facilitating memory, learning, and emotional regulation [40]. BDNF regulates neuronal synaptic plasticity (Lee et al. 2021) in the peripheral and central nervous systems by suppressing proinflammatory cytokines [41,42]. Fecal metabolites are associated with neurotransmission, complex cognitive behaviors, and BDNF function [43]. Based on our findings, we concluded that inosine regulates the BDNF and NGF levels, thereby contributing to improvements in D-galactose-induced cognition.

## 5. Conclusions

In summary, our study revealed that the oral administration of *L. plantarum* MWFLp-182 significantly regulated inosine levels in D-gal-treated BALB/c mice. The effect of inosine was potentially mediated by reducing the expression of IL-1β, TNF-α, TLR4, MyD88, and NF-κB; increasing IL-10, Nrf2, and HO-1 expression; and enhancing BDNF and NGF levels in the HT-22 cells. Consequently, inosine alleviated D-gal-induced oxidative damage, inflammation, and apoptosis in HT-22 cells. Based on these findings, future research should first delineate the origin of the elevated inosine and its upstream molecular targets, to fully elucidate the mechanism behind its coordinated regulation of the NF-κB and Nrf2 pathways. Furthermore, validating these neuroprotective effects in naturally aged animal models will be a crucial next step to confirm the anti-aging potential of this probiotic intervention. Our study elucidates the promising potential of *Lactiplantibacillus plantarum* MWFLp-182 as a novel dietary supplement or functional food ingredient. It is expected to be developed into a natural nutritional strategy to support brain health and combat oxidative stress-related neuronal aging.

## Data Availability

The original contributions presented in the study are included in the article/Supplementary Materials. Further inquiries can be directed to the corresponding author.

## Supplementary Materials

The following supporting information can be downloaded at https://www.mdpi.com/article/10.3390/foods15020349/s1, Table S1: Genes and primers selected for RT-qPCR; Table S2: Serum metabolite elution gradient of the mobile phase (positive ion mode); Table S3: Serum metabolite elution gradient of the mobile phase (negative ion mode); Table S4: Serum Metabolomics Mass Spectrometry Parameters; Table S5: Fecal metabolite elution gradient of the mobile phase; Table S6: Serum Metabolomics Mass Spectrometry Parameters.

## Abbreviations

The following abbreviations are used in this manuscript:

IL-10	Interleukin-10
IL-1β	Interleukin-1β
TNF-α	Tumour necrosis factor α
FBS	Fetal bovine serum
Nrf2	Nuclear factor erythroid 2-related factor
BDNF	Brain-derived neurotrophic factor
Bax	BCL-2-associated X
TLR4	Toll-like receptor 4
MyD88	Myeloid differentiation primary response protein 8
NF-κB	Nuclear factor kappa-B
HO-1	Heme oxygenase 1
ROS	Reactive oxygen species
NGF	Nerve growth factor
SCFAs	Short-chain fatty acids
D-gal	D-galactose
BBB	Blood–brain barrier
5-HT	Serotonin
MAPKs	Mitogen-activated protein kinases
BCAAs	Branched-chain amino acids.
