# Supplementary material for: Mechanism of Inosine from Lactiplantibacillus plantarum MWFLp-182-Treated Mice Model in Alleviating D-Galactose-Induced HT-22 Cell Injury via Oxidative and Inflammatory Pathways
Source: Foods. 2026 Jan 18;15(2):349. doi: 10.3390/foods15020349 (PMC12841473; doi:10.3390/foods15020349)
Supplement: Supplementary file 1 [file foods-15-00349-s001.zip › foods-4045658-supplementary.pdf]

Supplementary Information

Table S1. Genes and primers selected for RT-qPCR.

Gene	Primer	Sequence (5′ to 3′)
	Forward	TCATACTTCGGTTGCATGAAGG
BDNF	Reverse	AGACCTCTCGAACCTGCCC
	Forward	CCAGTGAAATTAGGCTCCCTG
NGF	Reverse	CCTTGGCAAAACCTTTATTGGG
	Forward	ATGGCATGGCTTACACCACC
TLR4	Reverse	GAGGCCAATTTTGTCTCCACA
	Forward	TCATGTTCTCCATACCCTTGGT
Myd88	Reverse	AAACTGCGAGTGGGGTCAG
	Forward	ATGGCAGACGATGATCCCTAC
NF-κB	Reverse	CGGAATCGAAATCCCCTCTGTT
	Forward	TGAAGACAGGGGCCTTTTTG
Bax	Reverse	AATTCGCCGGAGACACTCG
	Forward	TGGTGATGAAGGGGTCATTTATG
Caspase-3	Reverse	TTCGGCTTTCCAGTCAGACTC
	Forward	GACGCTCTGCTGAGTCGAG
Caspase-9	Reverse	GGTCTAGGGGTTTAACAGCCTC
	Forward	ATGCCTTTGTGGAACTATATGGC
Bcl-2	Reverse	GGTATGCACCCAGAGTGATGC
	Forward	TCTTGGAGTAAGTCGAGAAGTGT
Nrf2	Reverse	GTTGAAACTGAGCGAAAAAGGC
	Forward	AAGCCGAGAATGCTGAGTTCA
HO-1	Reverse	GCCGTGTAGATATGGTACAAGGA
	Forward	GACTTTAAGGGTTACCTGGGTTG
IL-10	Reverse	CTTACTGACTGGCATGAGGATCA

Supplement to Detailed Experimental Methods

1. Serum Metabolomic Analysis

The samples were tested by Shanghai Meiji Biotechnology Co., Ltd. The specific method was as follows: after collecting blood from mice, centrifuge (3500 × g, 4℃, 20 min), put the serum into a centrifuge tube, and quickly place it at -80 ℃ for testing. Sample pretreatment: Pipette 100 μL of sample into a 1.5 mL centrifuge tube, add 300 μL of extraction buffer (methanol:acetonitrile=1:1 (V:V) and internal standard (L-2-chlorophenylalanine (0.02 mg/mL)); vortex for 30 s, then sonicate for 30 min (5℃, 40 kHz); place the sample in a -20℃ freezer for 30 min; then centrifuge for 15 min (13000×g, 4℃), collect the supernatant, and dry it under nitrogen; add 100 μL of reconstitution solution (acetonitrile:water=1:1) to reconstitute; vortex for 30 s, then sonicate for 5 min (5℃, 40 kHz); centrifuge for 10 min (13000×g, 4℃), collect the supernatant, filter through a membrane into a vial for analysis. The LC-MS was performed using a Thermo Fisher Scientific UHPLC-Q Exactive HF-X system with tandem Fourier transform mass spectrometry. Chromatographic conditions: Column: ACQUITY UPLC HSS T3 (100 mm × 2.1 mm i.d., 1.8 μm; Waters, Milford, USA); Mobile phase A: 95% water + 5% acetonitrile (containing 0.1% formic acid); Mobile phase B: 47.5% acetonitrile + 47.5% isopropanol

+ 5% water (containing 0.1% formic acid); Injection volume: 3 μL; Column temperature: 40 °C. Liquid phase flow and mass spectrometry are shown in Tables S2, S3, and S4. The experiment was conducted in six parallel sessions.

Table S2. Serum metabolite elution gradient of the mobile phase (positive ion mode)

Time (min)	Flow Rate (mL/min)	A (%)	B (%)
0	0.4	100	0
3	0.4	80	20
4.5	0.4	65	35
5	0.4	0	100
6.3	0.4	0	100
6.4	0.4	100	0
8	0.4	100	0

Table S3. Serum metabolite elution gradient of the mobile phase (negative ion mode)

Time (min)	Flow Rate (mL/min)	A (%)	B (%)
0	0.4	100	0
1.5	0.4	95	5
2	0.4	90	10
4.5	0.4	70	30
5	0.4	0	100
6.3	0.4	0	100
6.4	0.4	100	0
8	0.4	100	0

Table S4. Serum Metabolomics Mass Spectrometry Parameters

Description	Parameter
Scan type（m/z）	70-1050
Sheath gas flow rate（arb）	50
Aux gas flow rate（arb）	13
Heater temp（℃）	425
Capillary temp（℃）	325
Spray voltage（+）（V）	3500
Spray voltage（-）（V）	-3500
S-Lens RF Level	50
Normalized collision energy（eV）	20,40,60
Resolution（Full MS）	60000
Resolution（MS2 ）	7500

2. Fecal Metabolomic Analysis

The samples were tested by Shanghai Lumin Biotechnology Co., Ltd., with slight modifications based on the references in the manuscript. The specific testing method was as follows: 60 mg of sample was weighed into a 1.5 mL centrifuge tube, two small steel balls and 600 μL of methanol-water (V:V=4:1 including mixed internal standard, 4 μg/mL) were added; after pre-cooling in a -40℃ refrigerator for 2 min, the sample was ground in a grinder (60 Hz, 2 min); after sonication in an ice-water bath for 10 min, the sample was allowed to stand overnight at -40℃, centrifuged for 10 min (13000×g, 4℃), and 200 μL of supernatant was collected and dried under nitrogen; the sample was reconstituted with 300 μL of methanol-water (V:V=1:4), vortexed for 30 s, sonicated in an ice-water

bath for 3 min, allowed to stand at -40℃ for 2 h, centrifuged for 10 min (13000×g, 4℃), and 150 μL was drawn up using a syringe. The supernatant was filtered through a 0.22 μm organic phase pinhole filter and transferred to a liquid chromatography vial, stored at -80°C until LC-MS analysis. The analytical instrument was a Waters ACQUITY UPLC I-Class plus/Thermo QE ultra-high performance liquid chromatography-tandem high-resolution mass spectrometer. Chromatographic column: ACQUITY UPLC HSS T3 (100 mm × 2.1 mm, 1.8 μm); column temperature: 45°C; mobile phase: A-water (containing 0.1% formic acid), B-acetonitrile; flow rate: 0.35 mL/min; injection volume: 2 μL. Liquid chromatography and mass spectrometry parameters are shown in Table S5 and S6. The experiment was conducted in six parallel sessions.

Table S5. Fecal metabolite elution gradient of the mobile phase

Time (min)	Flow Rate (mL/min)	A (%)	B (%)
0	0.35	95	5
2	0.35	95	5
4	0.35	70	30
8	0.35	50	50
10	0.35	20	80
14	0.35	0	100
15	0.35	0	100
15.1	0.35	95	5
16	0.35	95	5

Table S6. Serum Metabolomics Mass Spectrometry Parameters

Description	Parameter
Scan type (m/z)	100-1200
Sheath gas flow rate (arb)	35
Aux gas flow rate (arb)	8
Heater temp (℃)	425
Capillary temp (℃)	320
Spray voltage (+) (V)	3800
Spray voltage (-) (V)	-3000
S-Lens RF Level	50
Normalized collision energy (eV)	10,20,40
Resolution (Full MS）	70000
Resolution (MS2)	17500

3. Measurement of intracellular ROS in HT-22 cells

HT-22 cells were seeded in a 24-well plate at a density of 1×105 cells per well at 37 ℃ for 24 h. At 85% confluence, the cells were washed three times with PBS and HT-22 cells were treated with D-gal (0.5 mL; 20 mg/mL) and pretreated with inosine at different concentrations (0.5 mL, 0, 250, or 500 μg/mL) for 24 h. The cells were washed three times with PBS, and incubated with 500 μL of 10 mmol/L DCFH-DA in the dark at 37 ℃ for 30 min. Subsequently, the cells were washed twice with PBS and the fluorescence intensity was determined using fluorescence microscopy (OLYMPUS DP74, Japan) at excitation and emission wavelengths of 488 and 525 nm, receptively.

4. Determination of mRNA expression

RT-qPCR was used to quantify the mRNA expression levels of various factors, including the cytokines *IL-1β*, *IL-10*, *TNF-α*, *Nrf2*, *HO-1*, *Bax*, and *Bcl-2*. In addition, the expression levels of *AKT*, *Caspase-3*, *Caspase-9*, *MyD88*, *NF-κB*, and *TLR4* were also detected. *BDNF* and *NGF* expression were also assessed. Specific procedures: HT-22 cells were seeded in 24-well plates at a density of $1×10^5$ cells per well for 24 hours at 37℃. At 85% confluence, cells were washed three times with PBS, treated

with D-galactose (0.5 mL; 20 mg/mL), and pretreated with different concentrations (0.5 mL; 0, 250, or 500 μg/mL) of inosine for 24 h. Subsequently, HT-29 cells were collected and lysed, and total RNA was extracted according to the manufacturer's instructions (FastPure® Cell/Tissue Total RNA Isolation Kit V2, China). RNA concentration was determined using a microspectrophotometer (SpectraMax® QuickDrop™, USA), and purity was verified using A260:A280 and A260:A230 absorbance ratios. RNA reverse transcription was performed using the cDNA Synthesis kit (HiScript® IIQ RT SuperMix for qPCR (+gDNA wiper)), and qPCR was performed using a ChamQ Universal SYBR qPCR Master MIX (Vazume, China) on a StepOne real-time PCR system (Thermo Fisher Scientific, Applied Biosystems, USA). After qPCR, relative gene expression levels were calculated using the 2-ΔΔCT method. All genes and primers are shown in Table S1. The experiment was repeated three times, with three parallel experiments. The qPCR method was as follows:
Stage 1: 95°C for 30 s, 1 amplification cycle,
Stage 2: 95°C for 10 s, 60℃ for 30 s, 40 amplification cycles,
Stage 3: 95°C for 15 s, 60℃ for 60 s, 95°C for 15 s, 1 amplification cycle.

5. Immunofluorescence staining

HT-22 cells were seeded at a density of 5×10^4 cells per well in 12-well plates and cultured to 80% confluence. After trypsin digestion, cells were seeded again at a density of 5×10^4 cells/mL in 12-well plates. After washing three times with PBS, 0.5 mL of metabolite (final concentration 20 mg/mL, 0.5 mL) and 0.5 mL of D-gal (0.5 mL, 0, 250, or 500 μg/mL) were added, and the plates were cultured at 37℃ and 5% CO_2 for 24 h. Following treatment, cells were washed once with PBS, fixed with 4% paraformaldehyde at room temperature for 10 min, and then permeabilized with PBS containing 0.1% Triton X-100 for 1 h. Cells were blocked with 3% bovine serum albumin (BSA) in PBS for 1 h, followed by the addition of 300 μL of primary antibody against BDNF or NGF, and incubated overnight at 4℃ . Cells were then washed three times with PBS for 10 min each time, followed by incubation with FITC-labeled goat anti-rabbit IgG secondary antibody (1:400) at room temperature in the dark for 1 h. After secondary antibody incubation, cells were washed twice with PBS and mounted with 50 μL of mounting medium containing DAPI. Finally, cell nuclear staining was observed using a fluorescence microscope at an excitation wavelength of 330-380 nm and an emission wavelength of 420 nm, while the FITC-labeled secondary antibody was observed at an excitation wavelength of 488 nm and an emission wavelength of 500-540 nm. The experiment was conducted in six parallel sessions.
